# Cost-effectiveness of monitoring endoxifen levels in breast cancer patients adjuvantly treated with tamoxifen

**DOI:** 10.1007/s10549-018-4886-8

**Published:** 2018-07-13

**Authors:** M. van Nuland, R. A. Vreman, R. M. T. ten Ham, A. H. M. de Vries Schultink, H. Rosing, J. H. M. Schellens, J. H. Beijnen, A. M. Hövels

**Affiliations:** 1 Department of Pharmacy and Pharmacology, The Netherlands Cancer Institute and MC Slotervaart, Amsterdam, The Netherlands; 2 Division of Pharmacology, The Netherlands Cancer Institute, Plesmanlaan 121, 1066 CX Amsterdam, The Netherlands; 3 Division of Pharmacoepidemiology and Clinical Pharmacology, Utrecht Institute for Pharmaceutical Sciences, Utrecht University, Utrecht, The Netherlands; 4 Division of Clinical Pharmacology, Department of Medical Oncology, The Netherlands Cancer Institute, Amsterdam, The Netherlands

**Keywords:** Therapeutic drug monitoring, Cost-effectiveness, Tamoxifen, Endoxifen, Breast cancer

## Abstract

**Purpose:**

Breast cancer is the most common malignancy in women worldwide. Recurrence rates in breast cancer are considered to be dependent on the serum concentration of endoxifen, the active metabolite of tamoxifen. The goal of this study is to investigate the cost-effectiveness of periodically monitoring serum concentrations of endoxifen in adjuvant estrogen receptor alfa (ERα) positive breast cancer patients treated with tamoxifen in the Netherlands.

**Methods:**

A Markov model with disease-free survival (DFS), recurrent disease (RD), and death states was constructed. The benefit of drug monitoring was modeled via a difference in the fraction of patients achieving adequate serum concentrations. Robustness of results to changes in model assumptions were tested through deterministic and probabilistic sensitivity analyses.

**Results:**

Monitoring of endoxifen added 0.0115 quality-adjusted life-years (QALYs) and saved € 1564 per patient in the base case scenario. Deterministic sensitivity analysis demonstrated a large effect on the incremental cost-effectiveness ratio (ICER) of the differences in costs and utilities between the DFS and RD states. Probabilistic sensitivity analysis showed that the probability of cost-effectiveness at a willingness to pay of € 0 per quality-adjusted life-year (QALY) was 89.8%.

**Conclusions:**

Based on this model, monitoring of endoxifen in adjuvant ERα + breast cancer patients treated with tamoxifen is likely to add QALYs and save costs from a healthcare payer perspective. We advise clinicians to consider integrating serum endoxifen concentration monitoring into standard adjuvant tamoxifen treatment of ERα + breast cancer patients.

## Introduction

Breast cancer is the most common malignancy in women worldwide [1]. The heterogeneity of breast cancer manifests in a broad differentiation of phenotypes and morphological profiles. Breast cancer can be categorized, based on immunohistochemical features, into three main types: hormone receptor positive, human epidermal growth factor receptor 2 positive, and triple-negative tumors [2]. Hormone receptor positive types are characterized by a positive status of the estrogen and/or progesterone hormone receptor. Women with estrogen receptor alfa (ERα) positive breast cancer can be treated with tamoxifen; an anti-hormonal drug that blocks estrogen signaling by antagonizing the estrogen receptor [3]. Adjuvant tamoxifen treatment in ERα positive breast cancer reduces recurrence and mortality rates [4]. The reduction in breast cancer recurrence and in breast cancer associated death is shown after 1–2 years of adjuvant therapy with tamoxifen. These benefits increase after 5 years of tamoxifen intake [5]. Prolongation of tamoxifen treatment up to 10 years further decreases recurrence and mortality rates in a subgroup of patients [6, 7]. Tamoxifen can be prescribed for both premenopausal and postmenopausal women with breast cancer. In the postmenopausal setting, tamoxifen can be administered for 2–3 years in sequence before or after aromatase inhibitors [8], while only tamoxifen is given to premenopausal women [9].

Although tamoxifen reduces recurrence and mortality rates in a large group of patients, variable efficacy of tamoxifen therapy remains a major clinical challenge [5]. The anti-estrogenic activity of tamoxifen is limited. However, tamoxifen is rapidly converted into metabolites by CYP enzymes. Z-Endoxifen and (Z)-4-hydroxytamoxifen are the most active metabolites, of which endoxifen is most abundant and therefore most relevant for the anti-tumor effect. Endoxifen is formed through conversion by CYP2D6. Madlensky et al. were the first to describe a relationship between endoxifen serum concentrations and breast cancer survival in a retrospective study [10]. Patients with endoxifen levels above the reported threshold of 5.97 ng/mL had a better disease prognosis with a 26% lower recurrence rate than women with endoxifen concentrations below 5.97 ng/mL (HR 0.74; 95% CI 0.55–1.00). Integration of tamoxifen concentrations and concentrations of metabolites (Z)-4-hydroxytamoxifen and *N*-desmethyltamoxifen in an anti-estrogenic activity score demonstrated that endoxifen can serve as a proxy for the total anti-estrogenic effect of tamoxifen and metabolites [11]. Approximately, 80% percent of patients treated with standard dose tamoxifen reach these target concentrations of 5.97 ng/mL [10, 12]. Therefore, treatment optimization may improve outcomes for the remaining 20% of patients. To identify patients with an exposure below 5.97 ng/mL, endoxifen concentrations can be monitored after therapy initiation. Consequently, dose increase can be recommended to women with endoxifen levels below the threshold. The clinical practice of measuring drug concentrations to individualize drug dosing is called therapeutic drug monitoring (TDM). The pharmacokinetics of endoxifen are suitable for TDM, considering stable steady-state concentrations, low inter-occasional variability, and easy measurement in serum [13, 14].

The goal of this study is to assess the cost-effectiveness of monitoring endoxifen serum concentrations and subsequent personalized dosing of tamoxifen in patients with ERα-positive breast cancer treated with tamoxifen in the adjuvant setting in the Netherlands.

## Methods

In order to model the costs and benefits of endoxifen monitoring in patients with ERα positive breast cancer treated with tamoxifen in the adjuvant setting in the Netherlands, a Markov state transition model was constructed in Excel (Microsoft, Redmond, WA). In the Markov model, time spent by patients in disease states was modeled. A difference in time spent in certain disease states between two populations is modeled due to the effect of an intervention. The intervention endoxifen monitoring is compared with no TDM. Incremental costs and effects are thus attributed to the intervention, leading to an incremental cost-effectiveness ratio (ICER), which represents the added costs divided by the added QALYs due to the intervention. The ICER indicates how much should be invested to gain one QALY.

**Fig. 1:**
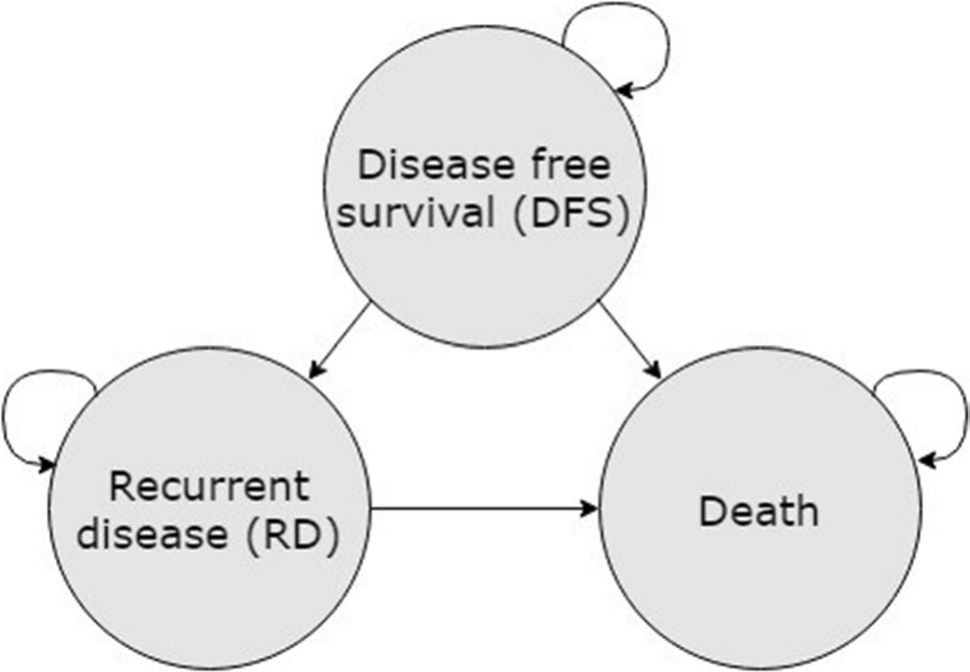
Markov model structure

The model included three disease states: disease-free survival (DFS), recurrent disease (RD), and death (Fig. 1). Approximately 7–8% of patients transit from DFS to RD get a locoregional recurrence [15, 16]. This type of recurrence can again be treated with tamoxifen [17]. No information is available on the probabilities of recurrence or death in this patient population. As we expect a higher probability of recurrent disease and death compared to patients with first-line tamoxifen treatment, we modeled these patients as staying in the RD state. Cycle duration was 28 days with an effectively lifetime horizon. Total quality-adjusted life-years and costs were calculated for adjuvant tamoxifen treatment with and without concomitant therapeutic drug monitoring of endoxifen serum concentrations. The analysis is performed from a healthcare payer perspective in The Netherlands. All input parameters and their ranges for sensitivity analyses are specified in Table 1. This method section is constructed according to the CHEERS reporting guideline [18].

**Table 1:** Input parameters and the ranges used in deterministic and probabilistic sensitivity analysis

Parameter	Base	Low	High	Distribution	Mean	SE	Source
Patient characteristics							
Age	53			Fixed	N/A	N/A	(18)
Discount rates							
Costs	0.045			Fixed	N/A	N/A	(28)
Effects	0.015			Fixed	N/A	N/A	(28)
Survival							
DFS (low endoxifen levels)							
Intercept	3.28	3.20	3.37	Lognormal (correlated)	N/A	N/A	(5, 10)
log(scale)	0.61	0.57	0.64	Lognormal (correlated)	N/A	N/A	(5, 10)
DFS (high endoxifen levels)							
Hazard ratio high versus low endoxifen	0.74	0.55	1.00	Lognormal	− 0.301	0.153	(10)
Breast cancer mortality (RD survival)							
Intercept	3.71	3.66	3.76	Lognormal (correlated)	N/A	N/A	(5, 20)
log(scale)	0.40	0.37	0.42	Lognormal (correlated)	N/A	N/A	(5, 20)
Endoxifen levels					Alfa	Beta	
% of patients high endoxifen at start	0.76	0.57	0.94	Beta	14.8	4.7	(19)
% of patients high endoxifen after dose increase	0.94	0.76	1.00	Beta	13.2	0.8	(19)
Annual costs							
DFS state	2872	1769	3975	Gamma	26.0	110.4	(M-C16)
RD state	16,125	9980	22,270	Gamma	26.5	609.5	(M-C16)
Death	8296	6222	10,370	Gamma	61.5	135.0	(M-C16)
Endoxifen blood level testing	113	85	141	Gamma	61.5	1.8	(22)
Utilities							
DFS state	0.80	0.73	0.87	Beta	99.55	24.89	(M-C16)
RD state	0.73	0.66	0.80	Beta	112.07	41.45	(M-C16)

### Modeled patients and intervention

The starting average age of patients was assumed equal to those found in the Women’s Healthy Eating and Living randomized trial (53 years), which was the source for the data on differences between recurrence rates for high and low endoxifen serum concentrations [10, 19].

The intervention consists of testing the serum concentration of endoxifen 3 months after starting treatment with tamoxifen, to ensure steady-state concentrations based on tamoxifen 7-day half-life [13]. Serum levels of endoxifen are defined as low when they are below 5.97 ng/mL and as high when they are equal to or above 5.97 ng/mL, according to the target defined by Madlensky et al. [10]. For all patients showing low serum concentrations, dosage of tamoxifen is doubled and their endoxifen serum concentration is evaluated after another 3 months. The percentage of patients that do not reach high serum concentrations of endoxifen was extracted from literature and found to be 24% after the first test and 6% after dose increased to 30 or 40 mg per day, as decided by the treating physician [20]. Decreasing endoxifen concentration after tamoxifen dose escalation was modeled as impossible.

Quality of life before and after dose increase of tamoxifen was assumed equal. This is based on the fact that no correlation has been found between adverse events, such as hot flashes, and serum concentrations of tamoxifen and its metabolites [21]. Furthermore, dose increase of tamoxifen in patients with reduced or absent CYP2D6 activity did not increase adverse events [22]. This suggests that tamoxifen dose can be increased while preserving quality of life.

### Survival estimates

Disease-free survival and breast cancer mortality were included from a meta-analysis on tamoxifen efficacy from the Early Breast Cancer Trialists’ Collaborative Group (EBCTCG) [5]. These estimates are based on 10,645 women with ERα-positive breast cancer treated with adjuvant tamoxifen for about 5 years.

Disease-free survival as estimated in the meta-analysis was first corrected for the hazard ratio provided by Madlensky et al. for patients with low versus high serum concentrations of endoxifen, namely, 0.74 (95% CI 0.55–1.00) [10]. To extrapolate survival curves beyond the duration of the EBCTCG trial, multiple parametric survival curves (exponential, Weibull, log-normal, log-logistic) were fitted on the published survival data for patients with a low serum concentration, according to the method provided by Hoyle and Henley [23]. This method appropriately reconstructs individual patient data from published curves. The best fit according to the Akaike Information Criterion (AIC) and Bayesian Information Criterion (BIC) was the lognormal curve. The lognormal curve is a function of an intercept and log(scale). The hazard ratio was applied to estimate disease-free survival for patients with high serum concentrations of endoxifen. The maximum hazard ratio that was possible in probabilistic analysis was 1.00. A lognormal curve also provided the best fit for breast cancer-related mortality. Overall survival (OS), assumed the same for both groups, was acquired by adding the Dutch national background mortality according to data from Statistics Netherlands (CBS), specified per age, to the breast cancer-related mortality provided by the meta-analysis [24]. Patients with recurrent disease represent the difference between overall survival and disease-free survival (RD = OS − DFS). Table 1 shows the used hazard ratio, and the intercept and log(scale) for the lognormal curves for disease-free survival and breast cancer survival. Survival rates for probabilistic sensitivity analysis are provided by a Cholesky correlation matrix according to the method provided by Hoyle and Henley [23].

### Cost and utilities

Costs are included from a Dutch health care perspective. Costs are discounted by 4.0% annually and presented in 2017 euros, as recommended by the Dutch National Health Care Institute (Zorginstituut Nederland, ZIN) [25, 26]. When disease costs were based on data from before 2017, cost inflation was performed with the Dutch national inflation calculator. Included costs are disease state costs and TDM costs. Costs for DFS and RD were included from a recent study on breast cancer costs for women with ERα-positive/HER2-negative breast cancer in The Netherlands [27, 28]. Mortality costs occurring at end-of-life are inflicted once in the cycle wherein death occurs. Drug monitoring costs are based on the tariff list of the Dutch Healthcare Authority [29]. All costs and ranges are specified in Table 1.

Utility values are implemented from the same study as disease state costs and are discounted by 1.5% annually, as recommended by ZIN [27, 30]. It was modeled as impossible for RD utility to be higher than utility in DFS. Utilities and used ranges are specified in Table 1.

### Sensitivity analysis

Deterministic and probabilistic sensitivity analyses were performed [31]. Deterministic sensitivity analysis showed the impact of varying each parameter individually according to its minimum and maximum value as specified in Table 1. This shows the importance of each individual parameter on incremental costs and QALY’s. Probabilistic sensitivity analysis included 10,000 trials with random values for all model parameters according to their individual distributions. Through randomly sampling all input parameters of the model simultaneously, a comprehensive estimate of the uncertainty around the model outcomes is provided. The model outcomes incremental costs, quality-adjusted life-years (QALYs), and the incremental cost-effectiveness ratio were calculated with a 95% confidence interval. Furthermore, a cost-effectiveness acceptability curve was calculated. This shows the likelihood that TDM is cost-effective (taking into account the uncertainty of the outcomes) in relation to different willingness-to-pay (WTP) thresholds, e.g., the probability that TDM is cost-effective if a decision-maker is willing to pay 20,000 euros for gaining one QALY.

### Clinical validation

As a clinical validation, we analyzed data from patients with ERα-positive breast cancer with tailored tamoxifen therapy in the adjuvant setting from the Antoni van Leeuwenhoek/ Netherlands Cancer Institute (AvL-NKI). Patients with endoxifen levels below 5.97 ng/mL and a dose increment were included. Serum samples were obtained as routine clinical care in the period between March 2013 and March 2017. Patients received tamoxifen at a dose of 20 mg. Dose escalation to 30 or 40 mg, as decided by the treating physician, was advised to patients with a serum concentration below 5.97 ng/mL and a second serum level was determined at least 3 months after dose adjustment. Endoxifen levels were measured with a validated liquid-chromatography mass spectrometry (LC-MS/MS) method [14].

**Fig. 2:**
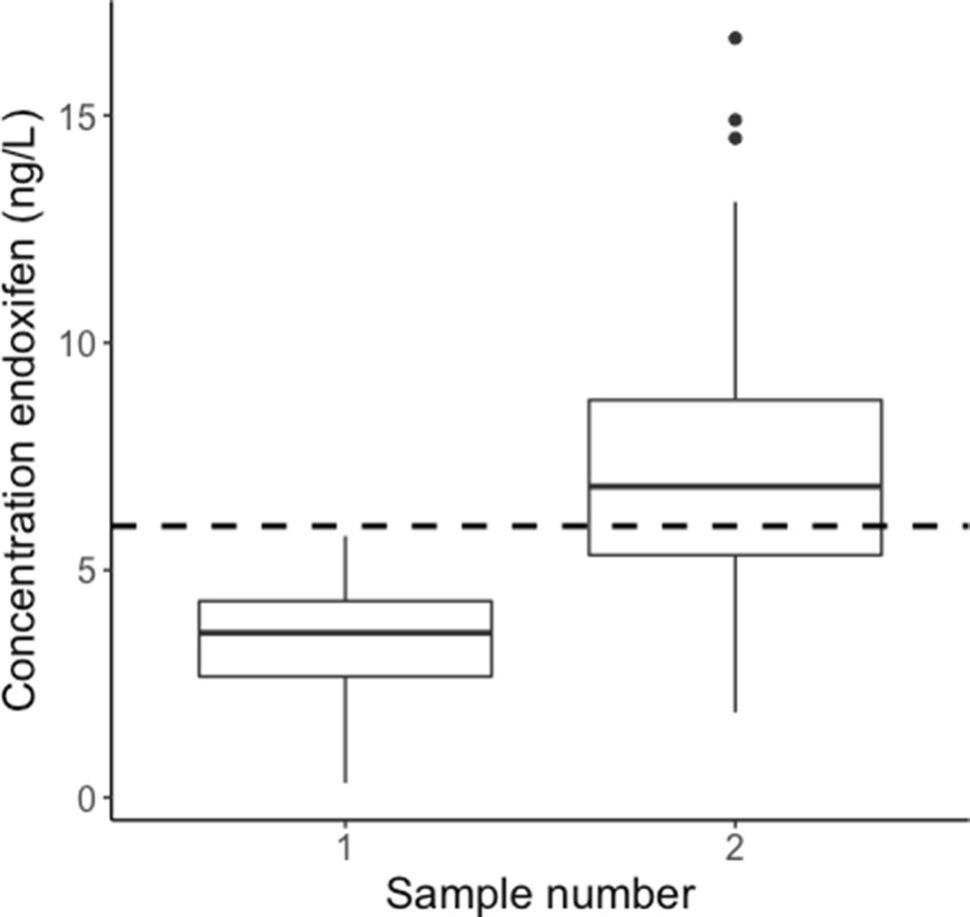
Boxplot showing endoxifen serum concentrations of 113 patients before (sampling point 1) and after (sampling point 2) dose increase. The dashed line represents the 5.97 ng/mL endoxifen threshold. After dose increase, 66.4% of patients have adequate serum levels

We found that of 813 patients for whom at least one serum test was available, 277 (34%) patients had a low serum concentration of endoxifen. From this cohort, we included 113 patients with a serum endoxifen level below 5.97 ng/mL to whom a dose increase was recommended. The remaining 164 patients were not evaluable because the tamoxifen dose was not increased after finding low endoxifen levels. Of 113 patients with low serum levels, a dose increment to 40 mg was advised to 90 patients and a dose increment to 30 mg was advised to 23 patients. In total, 66.4% of these patients reached the endoxifen target concentration of 5.97 ng/mL after dose increase (Fig. 2). This percentage is lower than previously reported. Jager et al. showed that 96% of patients reach the target concentration of 5.97 ng/mL after dose increase to 30 or 40 mg [32]. However, this was based on a small population of only 27 patients.

A low percentage of patients with adequate serum levels before dose increase leads to a potentially higher effect of monitoring. We therefore implemented the more conservative estimate from literature (76% in literature versus 66% from the clinical data [20]). For consistency, we have also included the previously reported percentage of patients that receive a dose increase that achieve adequate serum levels (75% in literature versus 66.4% from the clinical data). Furthermore, dose increases to 30 and 40 mg have been described. We chose to model dose increase to 40 mg to get a more conservative estimate of cost-effectiveness.

## Results

Base case results showed an overall reduction in costs of € 1,564 and an increase in QALYs of 0.0115 per patient due to therapeutic drug monitoring. Total average discounted costs for patients without TDM were € 48,809 and with TDM € 47,245. Total average QALYs without TDM were 15.32 and with TDM 15.33. This led to an ICER of € − 136,000.

**Fig. 3:**
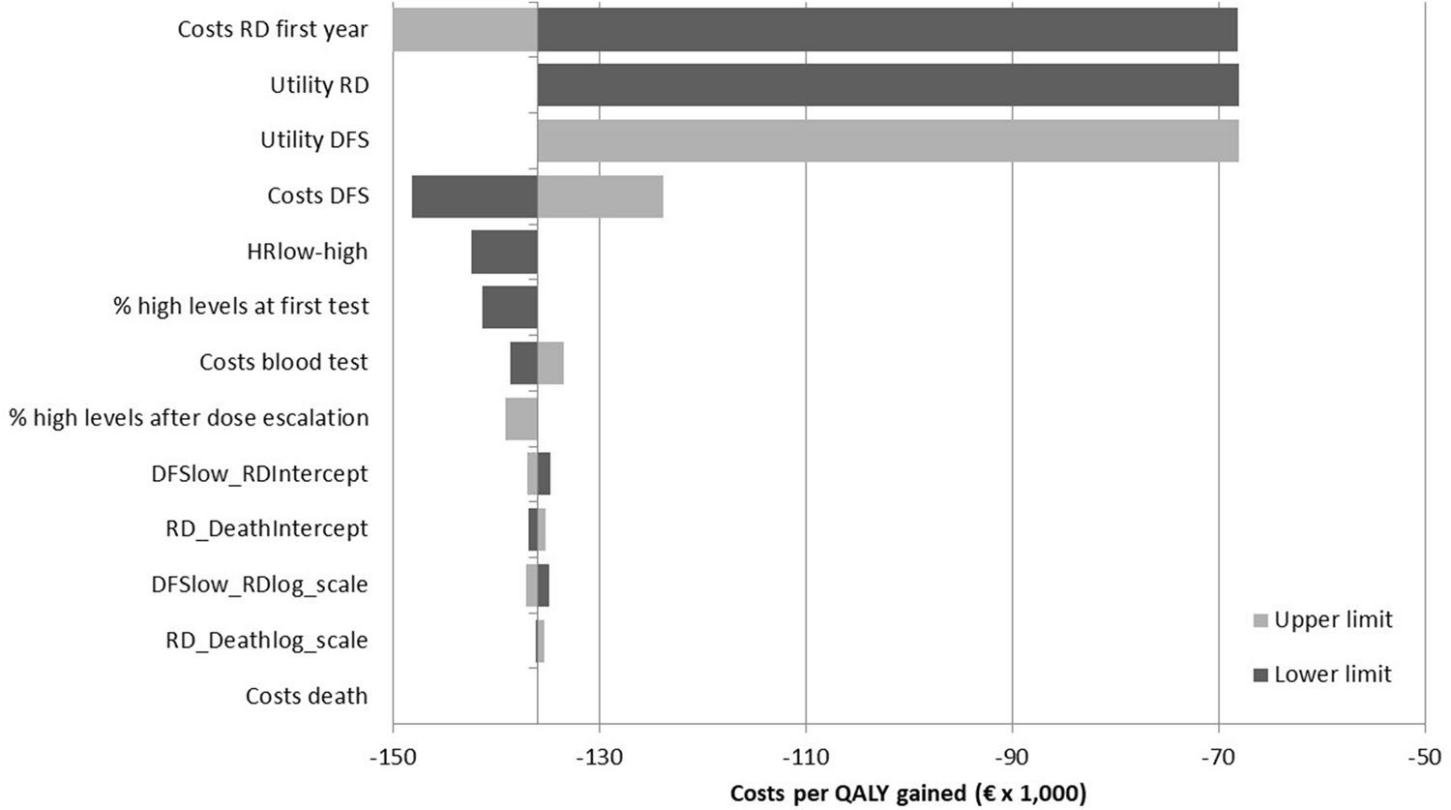
Deterministic sensitivity analysis. *TP* transition probability, *DFS* disease-free survival, *RD* recurrent disease, *low*/*high levels* low or high serum levels (cut-off 5.97 ng/mL) of endoxifen

The results from the one-way sensitivity analysis are shown in Fig. 3. Note that the ICER is negative due to the cost-saving effect of monitoring. No scenarios led to a positive ICER. However, five situations gave zero QALY benefit (ICER cannot be calculated). These situations represent extreme scenarios where there is no effect of drug monitoring: either there are no differences in utility between DFS and RD states, there is no effect of serum levels on recurrence (HR = 1.00), everyone already has a high serum concentration at the first test, or no one shows an increase in serum concentration after dose escalation.

**Fig. 4:**
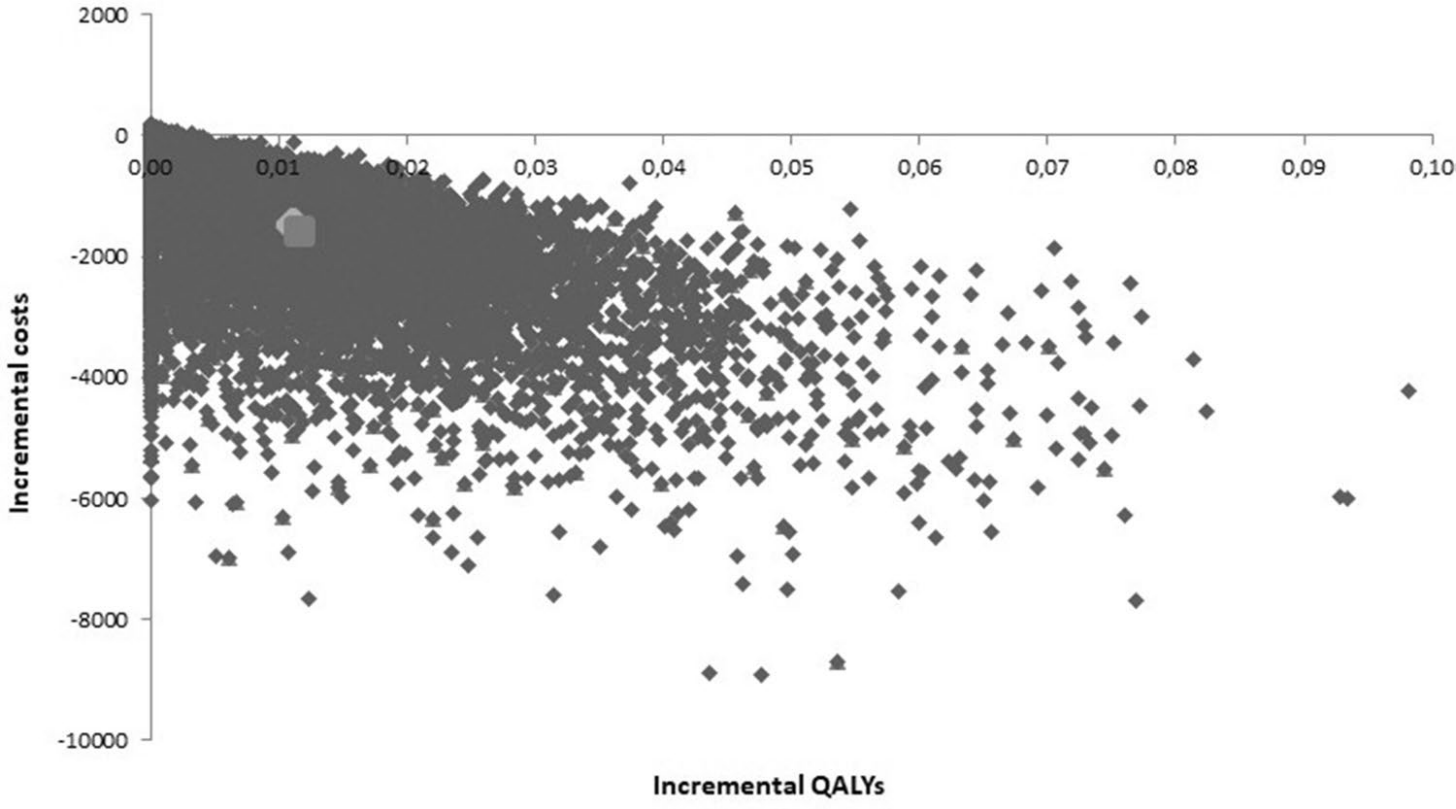
Results of the probabilistic sensitivity analysis in a cost-effectiveness plane. The larger light gray dot indicates the probabilistic mean and the larger darker gray dot indicates the base case scenario

The cost-effectiveness plane resulting from the probabilistic sensitivity analysis is shown in Fig. 4. Because the distribution of ICERs include two quadrants (upper and lower right), we cannot calculate a valid confidence interval around the mean ICER. A valid method to adequately show the uncertainty around the ICER is the calculation of incremental net monetary benefits by multiplying the incremental QALYs with the willingness-to-pay threshold and subtracting the incremental costs. We assumed a WTP threshold of € 20,000 per QALY as a conservative base case provided by the Dutch National Health Care Institute, with € 80,000 per QALY as an upper bound [26]. Mean net monetary benefits of serum monitoring were € 1687 (95% CI € − 133 to € 5089) for this base case WTP. The cost-effectiveness acceptability curve indicated that with a WTP of € 0 the probability of endoxifen serum concentration monitoring being cost-effective was 89.8%. This increased gradually to 90.6% with a WTP of € 80,000. It does not converge to 100% because of the inclusion of scenarios where no TDM benefit is demonstrated.

## Discussion

Our analysis shows that monitoring serum concentrations of endoxifen after 3 months and accordingly escalating the dose of adjuvant tamoxifen in women with breast cancer who have serum concentrations lower than 5.97 ng/mL will likely be cost effective. Indeed, in most cases this will be a cost-saving intervention. Just a minor intervention of one to two blood drawings will save an estimated € 1564 per patient. Though the individual QALY benefit is relatively small, the affected population is large which could lead to significant QALY’s gained on a macro level.

The difference between our intervention and our control arm is the number of people that will have a good serum concentration during DFS, which leads to a different distribution over DFS and RD states. Thus, the deterministic sensitivity analysis shows that inputs associated with these states, such as utilities and costs, have the biggest impact on the ICER. This is explained by the fact that if the utilities and costs in both living states converge, a big part of the effect is lost.

### Strengths and limitations

A strength of our approach is that it provides a straightforward and clinically supported way to estimate the cost-effectiveness of endoxifen serum concentration monitoring in breast cancer patients. Although the relationship between tamoxifen efficacy and endoxifen serum concentrations has been shown in a retrospective study, we are the first to show cost-effectiveness of monitoring during therapy optimization. Our conclusion of cost-effectiveness can guide best practices.

However, our analysis does have some limitations. First, the number of patients that acquire a serum concentration > 5.97 ng/mL after 3 months of treatment without dose escalation was extracted from a study with only 122 participants. We have validated these percentages by retrospectively assessing patient records in our hospital. Although the retrospective character of the clinical validation might cause selection bias of included patients, this is not a problem in our analysis as all patients with tamoxifen therapy were monitored. However, the Antoni van Leeuwenhoek hospital is a tertiary referral center, and patients visiting this hospital are referred for specialized healthcare. Despite the potential difference in study population, similar percentages were previously reported, and we believe that the clinical validation adequately describes the clinical setting. Additionally, we assumed that all patients with a low plasma concentration received a dose increase. In practice, this might not always be possible.

Additionally, our model is based on the underlying concept that a serum concentration lower than 5.97 ng/mL induces a higher risk for recurrence. Though this cut-off value is also used in clinical practice, it is based on a single retrospective analysis and thus might be subject to change when additional research is performed. Similarly, this study provided the hazard ratio (0.74) associated with these different recurrence risks. It would be best if the relationship between endoxifen serum concentrations and recurrence rates in the adjuvant setting was studied prospectively. Recent prospective trials studying the effect of endoxifen concentrations on clinical outcome were unable to confirm the 5.97 ng/mL threshold. However, in these studies endoxifen was monitored in neo-adjuvant and metastatic setting with a follow-up time of 5 years, which makes it difficult to properly interpret the relevance of the results for our study [33, 34]. Furthermore, power calculations were based on a rather large effect size, which might be less in real life. A threshold of endoxifen concentrations was not evaluated. To study the impact of varying the hazard ratio and the target threshold, we executed sensitivity analyses with the confidence interval of the described hazard ratio and the percentage of patients below or above the threshold. The deterministic sensitivity analysis shows that reducing these effects would still lead to a conclusion of cost-effectiveness (unless the effect is completely diminished). The probabilistic sensitivity analysis furthermore shows a high likelihood of cost-effectiveness. On the basis of these results, we would expect endoxifen monitoring to be cost-effective, even when differences between recurrence rates would be smaller.

## Conclusion

Based on this model, monitoring of endoxifen in adjuvant ER + breast cancer patients is likely to add QALYs and save costs from a healthcare payer perspective. We advise clinicians to consider integrating serum endoxifen concentration monitoring into standard adjuvant tamoxifen treatment of ERα + breast cancer patients.
